# Description of two measles outbreaks in the Lazio Region, Italy (2006-2007). Importance of pockets of low vaccine coverage in sustaining the infection

**DOI:** 10.1186/1471-2334-10-62

**Published:** 2010-03-11

**Authors:** Filippo Curtale, Fabrizio Perrelli, Jessica Mantovani, Marta Ciofi degli Atti, Antonietta Filia, Loredana Nicoletti, Fabio Magurano, Piero Borgia, Domenico Di Lallo

**Affiliations:** 1 Unit of Infecious Diseases and Vaccination, Department of Health Prevention and Promotion, Laziosanita' Agenzia di Sanita' Pubblica (ASP), Via Santa Costanza 53, 00198 Roma, Italy; 2 Unit of Epidemiology and Bio-Statistics, Healthcare Department, The Bambino Gesù Children's Hospital, Piazza S Onofrio 4, 00165 Rome, Italy; 3 National Centre for Epidemiology, Surveillance and Health Promotion (CNESPS), Istituto Superiore di Sanità (ISS), Via Giano della Bella 34 - 00162 Rome, Italy; 4 Department of Infectious, Parasitic and Immune-Mediated Diseases, Istituto Superiore di Sanità, Viale Regina Elena 299, 00161 Rome, Italy; 5 Scientific Directorate, Laziosanita' Agenzia di Sanita' Pubblica, Via Santa Costanza 53, 00198 Rome, Italy; 6 Department of Health Prevention and Promotion, Laziosanita' Agenzia di Sanita' Pubblica, Via Santa Costanza 53, 00198 Rome, Italy

## Abstract

**Background:**

Despite the launch of the national plan for measles elimination, in Italy, immunization coverage remains suboptimal and outbreaks continue to occur. Two measles outbreaks, occurred in Lazio region during 2006-2007, were investigated to identify sources of infection, transmission routes, and assess operational implications for elimination of the disease.

**Methods:**

Data were obtained from several sources, the routine infectious diseases surveillance system, field epidemiological investigations, and molecular genotyping of virus by the national reference laboratory.

**Results:**

Overall 449 cases were reported, sustained by two different stereotypes overlapping for few months. Serotype D4 was likely imported from Romania by a Roma/Sinti family and subsequently spread to the rest of the population. Serotype B3 was responsible for the second outbreak which started in a secondary school. Pockets of low vaccine coverage individuals (Roma/Sinti communities, high school students) facilitated the reintroduction of serotypes not endemic in Italy and facilitated the measles infection to spread.

**Conclusions:**

Communities with low vaccine coverage represent a more serious public health threat than do sporadic susceptible individuals. The successful elimination of measles will require additional efforts to immunize low vaccine coverage population groups, including hard-to-reach individuals, adolescents, and young adults. An enhanced surveillance systems, which includes viral genotyping to document chains of transmission, is an essential tool for evaluating strategy to control and eliminate measles

## Background

The World Health Organization Regional Office for Europe (WHO/EURO) has set 2010 as the target for elimination of measles in the region [1,2]. This objective has already been achieved in some States through routine immunization programmes which maintain high coverage using a two-dose measles-mumps-rubella (MMR) vaccine schedule [3]. However, vaccination coverage still remains inadequate in several western European countries, including Italy, and although mass vaccination has successfully lowered the incidence of the disease outbreaks continue to occur, often affecting communities with low vaccination coverage [4].

In Italy, measles vaccine (MV) was introduced in 1976 and routine administration of one dose of measles vaccine to all children aged ≥15 months was recommended since 1979. Combined MMR vaccines became available in the early 1990s and the two-dose schedule (first dose at 12-15 months and second dose at 5-6 or 11-12 years) was introduced in 1999 [5]. However, despite the existence of national recommendations, responsibility for implementation of measles vaccination activities lies within each of Italy's 21 regions. This has contributed to non uniform coverage across regions, with lower rates observed mainly in central and southern regions (including Lazio) with respect to northern regions [6].

In the years 2002-2003 a large measles outbreak occurred in Italy with over 100,000 estimated cases among children below 15 years of age [7]. Following the outbreak, in November 2003, all 21 Italian regions signed an agreement with the Italian Ministry of Health to implement the "National Plan for the Elimination of Measles and Congenital Rubella" [8]. Strategies include among others, improving routine MMR coverage among children below 2 years of age, implementing supplementary vaccination activities for older children and adolescents (aged 6-13 years) and strengthening disease surveillance. Following implementation of the elimination plan, national vaccination coverage for first MMR dose in children at two years of age increased from 82.0% in 2003, to 89.6% in 2007. During the same period of time, incidence of measles in Italy decreased from 22.6/100,000 to <1/100,000 [4,9].

In the Lazio region, the measles elimination plan succeeded in increasing coverage for the first dose of MMR among children at two years of age from 83.9% in 2003 to 90.7% in 2007 [10]. During the same five year period, coverage among school-aged children (6-13 years of age) increased from 53% to 67% while coverage for the second dose at 15 years of age increased from 14% to 42%. In 2004 and 2005 a historically low incidence of < 1 case per 100,000 was reached in the Lazio region, with approximately 50 measles cases reported per year. However, sporadic cases continued to be reported, especially among susceptible adolescent and adults. Pockets of low coverage were also present in specific areas and among emarginated and hard-to-reach populations (HRP), such as the Roma/Sinti population, and illegal immigrants [11].

Between June 2006 and August 2007, two measles outbreaks occurred in the Lazio region. The first outbreak (June-December 2006) initially involved the Roma/Sinti population, and subsequently spread to the rest of the population. The second outbreak overlapped with the first (October 2006-August 2007) and affected mainly the Italian adolescent and adult population. In this article we describe the two outbreaks and highlight the importance of pockets of low vaccine coverage in sustaining such outbreaks. Data from the mandatory infectious diseases surveillance system, field epidemiological investigations, and molecular characterization of measles virus by the national reference laboratory are presented.

## Methods

### Regional setting

Lazio, one of Italy's 21 regions, has a population of 5.3 million people (2006), 3.2 million of which living in the urban area of the capital city of Rome. It is divided into 5 provinces (Rome, Rieti, Viterbo, Latina and Frosinone) and 12 Local Health Units (LHUs).

### Data collection and case definition

The Public Health Agency of the Lazio Region *(Agenzia di Sanita' Pubblica, ASP)*, is responsible for surveillance of infectious diseases and immunisation coverage in the region. The ASP monitored the two measles outbreaks and coordinated outbreak control activities in the 12 LHUs of the region.

In Italy, measles is a disease subject to mandatory notification, and according to the routine procedure, physicians must report suspected measles cases to their LHU within 48 hours of diagnosis. The local health authorities then report confirmed measles cases to the ASP monthly. At the beginning of the outbreaks this procedure was modified and physicians were asked to report suspected measles cases to both the local health authorities and ASP offices within 24 hours of diagnosis. Personnel of the LHUs performed epidemiological investigation of suspected cases including laboratory investigation and contact tracing.

A suspected measles case was defined as a subject with fever (≥38°C), generalised rash and at least one of the following symptoms: cough, coryza, or conjunctivitis. A confirmed case was defined either as a laboratory confirmed case (in which measles-specific IgM antibodies were present in serum or saliva samples or measles virus nucleic acid was detected in urine samples by PCR or as a case with an epidemiological link to a confirmed case.

For each confirmed case, demographic data characteristics (including whether cases belonged to a Roma/Sinti community), vaccination history, date of onset of symptoms, and hospitalisation, were collected. Information was conveyed to the ASP, which discarded non confirmed cases from the database, eliminated redundant records, performed quality check and contacted the local health authorities for any missing information. To assess presence of indigenous transmission or sources of imported virus the data set was integrated with information provided by the National Institute of Health (*Istituto Superiore di Sanità*, ISS), which conducted viral molecular characterization from urine samples of measles cases, utilising the PCR technique [12]. Data analysis regarding the age distribution and immunization status of cases, as well as the percentage of cases requiring hospital admission was performed on the total number of cases reported in the two outbreaks.

### Analysis of measles sequence

Phylogenetic analysis based on the available partial nucleoprotein gene sequences of measles virus and tree reconstructions were performed with MEGA software version 4.0 [13]. Virus isolates and genotypes were designated according to the new official WHO nomenclature [14].

### Data management

Data from confirmed cases was entered into Microsoft Excel and converted to SAS version 8 (SAS Institute Inc., Cary, NC) for analysis. The temporal and geographical distribution of cases, together with the age distribution, was calculated separately for the Roma/Sinti and rest of the population.

### Ethics

The present study did not require approval from an Ethics Committee. Laziosanità - the public health agency of the Lazio Region is the local government agency responsible for the collection of infectious disease notifications, hospital admission and discharge records and laboratory surveillance data. The management of these data for public health purposes does not require a patient's informed consent nor does it require any authorization regarding privacy laws.

## Results

From June 2006 to the end of August 2007, a total of 449 cases were reported, of which 302 in 2006 and 147 in 2007. The two outbreaks overlapped and not all cases were genotyped; therefore, it was not possible to determine the exact number of cases that occurred in each of the two outbreaks. Overall, 347/449 cases occurred amongst the Italian ethnic population and 102/449 amongst the Roma/Sinti population. Seventy-eight cases (17%) were laboratory confirmed while the remaining were epi-linked. The virus serotype was identified for fifty-seven cases (Serotype D4: n = 32; serotype B3: n = 25).

### Description of outbreaks

#### Outbreak 1 (June-December 2006)

The first reported case was an eight year-old Roma child of Romanian nationality, living in a settlement located in the outskirts of Rome. The child was admitted to hospital with rash, fever, diarrhoea, conjunctivitis, rhinitis and otitis on 28 June 2006, reporting onset of symptoms on 24 June 2006. Analysis of routine data allowed the identification of an additional case that had been notified one week previously, in Rome by a different LHU. This case was an unvaccinated six-year-old Roma child, also of Romanian nationality, who developed symptoms on 16 June 2006. The child was not hospitalised and the source of his infection was not determined, since his parents refused to answer to questioning by the health officials and then moved away. He transmitted the infection to an eight-year-old child living in the same building, who developed symptoms on 1 July 2006 and was hospitalised. In the last week of June two additional Roma/Sinti children, in two different health authorities, developed measles symptoms on 23 June and 24 June respectively. By the end of July in Rome, an additional 25 measles cases had been reported in various settlements of the Roma/Sinti community and 16 cases among the rest of population. Two cases among the Italian ethnic population, who developed symptoms on 19 and 20 July, reported contact with Roma/Sinti patients with measles in a hospital waiting area, on 4 and 10 July 2006 respectively.

**Figure 1:**
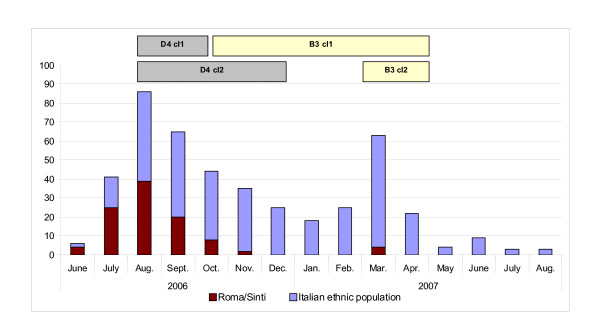
**Number of reported measles cases by month of onset of symptoms, amongst Rom/Sinti and Italian ethnic population, in the Lazio region, June 2006 - August 2007 (Total n = 449), and period of detection of different stereotypes**. In the boxes the sequence of different stereotypes and clusters of common origin identified. The two cases reported among the Italian ethnic population in the first two weeks of June, and the six cases in July-August 2007, should be considered endemic cases.

As of the end of November 2006, 98 cases had been reported in the Roma/Sinti community from 19 settlements in Rome. Approximately half of these cases (50/98) were of Romanian nationality. No further cases among the Roma/Sinti population were reported after November 2006. At the same time, a total of 204 cases were reported among the general population in Rome, from July to December 2006 (Figure 1).

#### Outbreak 2 (October 2006 - August 2007)

In November 2006, a cluster of six measles cases was reported amongst adolescents and young adults attending a professional school in the outskirts of Rome (attack rate 0.7%). The infection subsequently spread outside the school and cases continued to be reported amongst the Italian ethnic population until the summer 2007. This second outbreak reached a peak in March 2007 and was considered over only in August 2007 (Figure 1). During July-August 2007 three measles cases were notified per month, bringing the incidence level to that reported in the Lazio region before the described outbreaks (≤4 cases per month, or <1/100,000 population per year).

#### Molecular Characterization

The D4 genotype, grouped in two different clusters of common origin, was responsible for the first cases reported in the Roma/Sinti population and detected in several other cases, including the Italian ethnic population, up to December 2006. Starting in October 2006 the B3 genotype was isolated in a contact of a case from the school outbreak, overlapping for some time with genotype D4 (Figure 1).

#### Geographical distribution of the outbreaks in Lazio region

**Figure 2:**
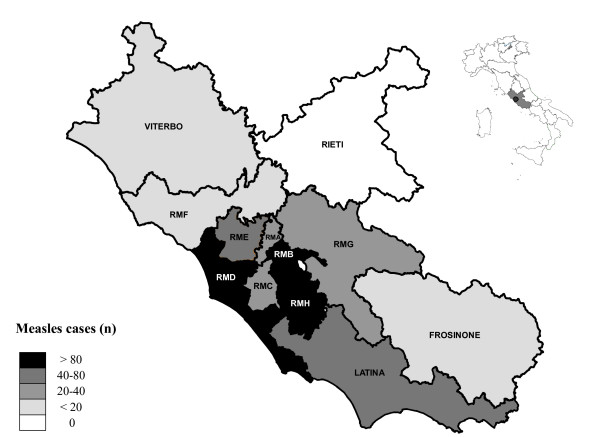
**Distribution of measles cases by district (LHU) of residence, in Lazio region, reported between June 2006 and August 2007 (Total n = 449)**. Urban Rome: LHU RM-A to RM-E. Province of Rome: RM-F to RM-H. 14 cases were diagnosed in Lazio, but resident in other regions.

The first outbreak (D4) started in the RM-B LHU (Figure 2). Cases were subsequently reported in a different area of Rome (RM-D) with numerous Roma/Sinti settlements. These two areas were the most affected by the outbreak while a more limited number of cases were reported in the remaining three health districts of the city of Rome (RM-A, RM-C, RM-E). Districts outside the urban area of Rome reported only sporadic cases, with the exception of district RM-H in the south of Rome, where the outbreak in the professional school occurred. (Figure 2).

#### Age distribution of the cases

**Figure 3:**
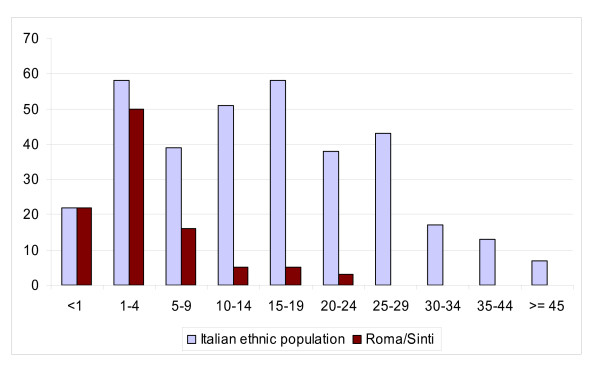
**Number of reported measles cases by age group, Roma/Sinti and Italian ethnic population**. Lazio region, June 2006 - August 2007 (n = 449).

Overall, the median age of cases was 11 years. Children aged 0-4 years were the most affected age group, with one third (152/449) of cases reported belonging to this age group (76/100,000 incidence for children <5 years). Measles incidence in the total population during the two outbreaks was 9/100,000. When analysed separately, the age distribution of measles cases was different among the Roma/Sinti population (median age two years) with respect to the rest of the population (median age 15 years). Seventy percent (72/102) of Roma/Sinti cases occurred in children aged 0-4 years and over 90% (93/102) were aged below 15 years. The age distribution of cases in the Italian ethnic population was more evenly distributed among all age groups. Only 23% of cases (80/347) were aged 0-4 years, and less than 50% (170/347) occurred in children below 15 years of age (Figure 3).

#### Immunization status, hospital admission, and nosocomial transmission

None of the Roma/Sinti cases were vaccinated against measles, and only 16 (5.5%) cases from the Italian ethnic population had received one dose of measles-containing vaccine. Overall, over 57% (258/449) of cases required hospital admission. Fifteen cases (3.3%), 12 of whom from the first outbreak and three from the second, reported a hospital contact with children affected by measles, either in the waiting area or after admission for a different condition. Four of these contacts were healthcare professionals (two nurses and two physicians). Out of the 15 reported cases of nosocomial transmission, seven involved contacts with Roma/Sinti children, including the first two cases in the Italian ethnic population.

#### Control measures

In response to the described clusters of measles cases, active tracing and vaccination of susceptible contacts was performed by local health authorities. A second dose of MMR vaccine was also offered to incompletely vaccinated contacts. In addition, MMR vaccine was offered to all susceptible or incompletely vaccinated children and adolescents attending any of the schools in which cases had been detected and to Roma/Sinti children up to 5 years of age. Vaccination sessions were conducted directly in the involved settlements; in total 493 persons in seven settlements were vaccinated in August 2006. Isolation of cases and susceptible contacts (in hospital for Roma/Sinti patients and at home for other subjects) was recommended,

Local health authorities were urged to identify possible contacts of all suspected measles cases, and alert general practitioners, family paediatricians, and hospitals about the outbreaks. Physicians were asked to report cases within 24 hours of diagnosis. In addition, guidelines regarding respiratory isolation of patients with suspected measles, and vaccination of susceptible hospital staff were forwarded to all hospitals of the region. Before the start of the 2006/07 school year a meeting was organized by the ASP with staff in charge of the measles elimination campaign and the public health departments of the 12 health authorities. Local media were also informed of the outbreaks.

## Discussion

The two described outbreaks, which involved 449 cases (incidence 9/100,000) notified from June 2006 to August 2007, represent the most serious episodes after the 2002-2003 measles outbreak [7]. They confirmed that pockets of low vaccination coverage exist in some areas of the Lazio region, particularly among Roma/Sinti communities and adolescents

Thanks to relatively high immunisation rates amongst new born children (90,7%) [10] and the work done by the local health authorities, conducting contact investigation of cases', vaccination of susceptible school and household contacts, and implementing isolation measures, the outbreaks did not affect the whole region and, in the city of Rome, was mainly limited to a few peripheral districts (Figure 2). Both outbreaks started in populations known to have low coverage, (Roma/Sinti community, and students of a secondary school). The subsequent spread to the rest of the population, at least for the first outbreak, was facilitated by nosocomial transmission.

Differences were found in the affected age groups among the Roma/Sinti and the rest of population. As expected in a susceptible population, the most affected age group in the Roma/Sinti population was the 1-4 year-old age group. Conversely, in the Italian ethnic population, which had a higher percentage of vaccinated subjects with respect to the Roma/Sinti population, especially among young children, most cases occurred in the 15-19 year-old age-group.

Molecular characterization of measles virus is an important surveillance tool for monitoring measles elimination [15,16]. In this case it was fundamental in tracing the origin of both outbreaks and showing that two distinct chains of transmission took place in the region. It is highly likely that the first outbreak, due to the D4 measles serotype, which is not endemic in Italy [12], was imported from Romania. In fact, first cases occurred in families of Romanian nationality with family and social links in Romania. In addition, the D4 sequence identified among the first cases in Lazio was found to be identical to the D4 isolated in Romania during the 2004-2006 outbreak [17] in which over 4000 cases and 10 deaths occurred [18].

**Figure 4:**
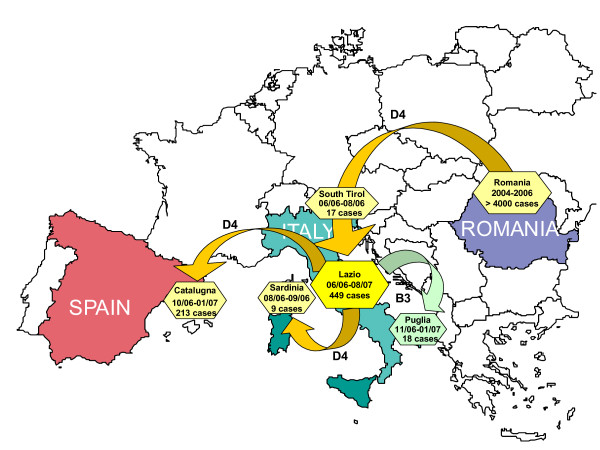
**Measles outbreaks reported in Southern Europe in the years 2006-2007**. Diffusion of the two measles genotypes affecting Lazio. Duration of the outbreaks in months and number of subjects affected in each outbreak are indicated in the boxes.

A D4 measles strain highly correlated with the one isolated in Lazio caused a smaller outbreak in northern Italy (South Tyrol), between 21 June and 11 August 2006 [19]. The first case was reported during the same week of the beginning of the first outbreak in Lazio, and 13 of 17 cases belonged to the Roma/Sinti population. In South Tyrol, a transit camp for Roma/Sinti travelling between Italy and Eastern Europe appeared to be the entry point for the D4 measles genotype in Italy (Figure 4).

Between August 2006 and February 2007 other measles outbreaks linked to the Lazio outbreak (D4) occurred in Sardinia (Italy) and Barcelona (Spain), both affecting mainly Roma/Sinti communities. In Sardinia, nine cases, all aged below 15 years and three of which laboratory confirmed, with genotype D4 isolated, were reported from a Roma/Sinti settlement in the town of Alghero, between 12 August and 3 September 2006 [19]. Four of the children had travelled to Rome from 3 to 14 August 2006, to attend a funeral. In the Barcelona region (Spain) an outbreak occurred from October 2006 to February 2007. The first case, a six-year-old girl of Eastern European origin had attended a family gathering in Italy with her mother where other guests may also have had measles. Genotype D4 was identified [20] confirming the link with the outbreak in Lazio (Figure 4).

Genotype D4 was no longer isolated after the end of 2006, being replaced by B3 genotype starting in October 2006. Despite an accurate epidemiological investigation, the origin of this genotype was not identified. B3 is not considered endemic in Italy and is most frequently detected in Sub-Saharan Africa, although transmission of this virus within Europe has been reported. The isolated B3 was similar, but not identical, to the strain circulating in UK in 2005 [12]. This serotype was first identified in Lazio and subsequently introduced in Puglia (Figure 4), a region of South Italy, where it was responsible for an outbreak from November 2006 to January 2007 [21].

The percentage of cases which required hospitalisation during the two outbreaks in Lazio was high (57%). This can be partly explained by the well known underreporting of cases by general practitioners and paediatricians, as compared to hospital physicians. The number of cases requiring hospitalisation, especially during July-September 2006, was sufficiently high to create problems to the hospital system. The inadequate number of isolation beds in hospitals may represent a serious problem in case of occurrence of an epidemic due to a more aggressive infective agent, such as SARS or pandemic flu.

Measles nosocomial transmission has been recently documented in several other outbreaks in Italy and other European countries [22-25] and the public health importance of nosocomial measles transmission has been established in many situations.

The 15 cases reported in this paper represent only 3% of the total number of cases and therefore did not contribute significantly to the measles incidence during the outbreak. However, these cases most likely represent an underestimate of the real number of infections that occurred through hospital contacts and nosocomial transmission. It is likely that isolation measures and separate admission procedures were not always adopted in case of admission of a patient with signs and symptoms compatible with measles. In the first outbreak, nosocomial transmission may have been responsible for the spread of the infection from the Roma/Sinti to the Italian population. Also of concern is the fact that four healthcare professionals developed measles. As the incidence of measles declines, nosocomial transmission is likely to become an important source of infection and sustain the occurrence of outbreaks among non-immunised health staff and hospital contacts, representing a serious problem in the elimination of measles [26]. Strategies to minimize nosocomial spread of infection should become a priority for control and effectively implemented in the future.

## Conclusions

The described outbreaks highlight the threat represented by pockets of susceptible populations, even in the presence of good coverage levels in the overall population [27]. These groups include hard-to-reach populations (HRP) such as the Roma/Sinti communities (estimated MMR coverage in Italy below 10%,) [11], families who refuse vaccination for ideological or religious reasons, as reported in recent outbreaks amongst students in private religious schools and orthodox communities in Europe and Israel [28-30], and families objecting to having their children vaccinated out of concern for vaccine-related adverse events [31-33]. The risk for the community represented by HRP or organized groups of objectors should not be underestimated and represent a more serious treat than sporadic susceptible individuals. Susceptible population groups may reintroduce indigenous measles virus transmission even in countries confirmed as disease-free or in a population with high immunization coverage [34] facilitating the transmission of the disease to susceptible individuals still present in the region.

Efforts are needed to improve methods to identify areas with low coverage and to develop specific strategies which target susceptible groups. An improvement in health services delivery may be needed to reach Roma/Sinti communities and new immigrants. At the same time, more effective communication strategies should be defined to address subjects objecting to vaccination either for religious or other reasons, involving these subjects in a wider discussion on their responsibility toward the community. The implementation of catch up campaigns targeting adolescents and young adults should also be considered, with the additional objective of protecting women of childbearing age against rubella.

## Competing interests

The authors declare that they have no competing interests.

## Authors' contributions

FC coordinated the epidemiological data collection and the outbreaks' control measures, supervised the data analysis, and drafted the manuscript. FP supervised the local health units personnel and the implementation of public health measures. JM performed the statistical analysis and produced the graphs. MCdA revised the results of data analysis and contributed in drafting the manuscript. AF contributed to epidemiological data collection, participated in drafting and revising the manuscript. LN supervised the laboratory work and molecular genetic study. FM Carried out the sequence alignment, the molecular genetic studies and contributed in drafting the manuscript. PB formulated the original study hypothesis and participated in the study design. DDL conceived the study, and participated in its design and coordination. All authors read and approved the final manuscript

## Pre-publication history

The pre-publication history for this paper can be accessed here:

http://www.biomedcentral.com/1471-2334/10/62/prepub
